# A Nonsense Variant of *ZNF462* Gene Associated With Weiss–Kruszka Syndrome–Like Manifestations: A Case Study and Literature Review

**DOI:** 10.3389/fgene.2022.781832

**Published:** 2022-02-07

**Authors:** Shaozhi Zhao, Chen Miao, Xiaolei Wang, Yitong Lu, Hongwei Liu, Xinwen Zhang

**Affiliations:** Center of Medical Genetics, Xi’an People’s Hospital (Xi’an Fourth Hospital), Xi’an, China

**Keywords:** *ZNF462* gene, Weiss–Kruszka syndrome, ptosis, hearing loss, craniofacial deformities

## Abstract

**Objective:** This study aims to explore the clinical characteristics and genetic basis of a patient with unilateral ptosis and unilateral hearing impairment in pedigree analysis.

**Methods:** The clinical data of the child and his father were collected. The genomic DNA of the patient and his relatives were extracted from their peripheral blood samples and subjected to trio-whole-exome sequencing (trio-WES) and copy number variation analysis. Sanger sequencing was used to verify the potential variant.

**Results:** The sequencing analysis identified a heterozygous nonsense variant c.6431C > A (p.Ser2144*) in the *ZNF462* gene (NM_021224.6) in the child and his father, whereas the locus in his asymptomatic mother, brother, and grandparents was found to be the wild type, which is an autosomal dominant inheritance. The new genetic variant has not been previously reported in the ClinVar and HGMD databases and the Genome Aggregation Database (gnomAD).

**Conclusion:** This is the first incidence of Weiss–Kruszka syndrome relating to the nonsense variant in the *ZNF462* gene in China. The finding from this study is novel in its expansion of the variant spectrum of the *ZNF462* gene and clarifies the genetic etiology of the patient and his father.

## 1 Introduction

Weiss–Kruszka syndrome (WSKA, MIM: 618,619) is a multiple congenital anomaly syndrome. WSKA is characterized by ptosis, growth restriction, craniofacial deformities, and corpus callosum hypoplasia (Weiss et al., 2017). Recent evidence revealed that WSKA is caused by the loss-of-function (LOF) variations in the *ZNF462* gene or deletions on chromosome 9 p 31.2 containing the *ZNF462* gene. Additionally, this genetic disease is inherited in an autosomal dominant manner, which often results from new variants. So far, only 27 cases of *ZNF462* gene variation have been reported globally (Weiss et al., 2017; Cosemans et al., 2018; Kruszka et al., 2019; González-Tarancón et al., 2020; Iivonen et al., 2021; Park et al., 2021), and the underlying mechanism of the syndrome has not been extensively studied. This study reported a family in which both the child and his father had WSKA with ptosis and hearing loss. The diagnosis was established based on clinical symptoms and gene tests. Through trio-whole-exome sequencing (trio-WES), a novel nonsense variant in the *ZNF462* gene was identified in the child and his father. With the first pedigree analysis of WSKA in China, the study enriched the variant spectrum of the *ZNF462* gene and enhanced the knowledge of clinical features, genetic characteristics, and diagnostic protocols for WSKA.

## 2 Subjects and Methods

### 2.1 Subjects

**FIGURE 1:**
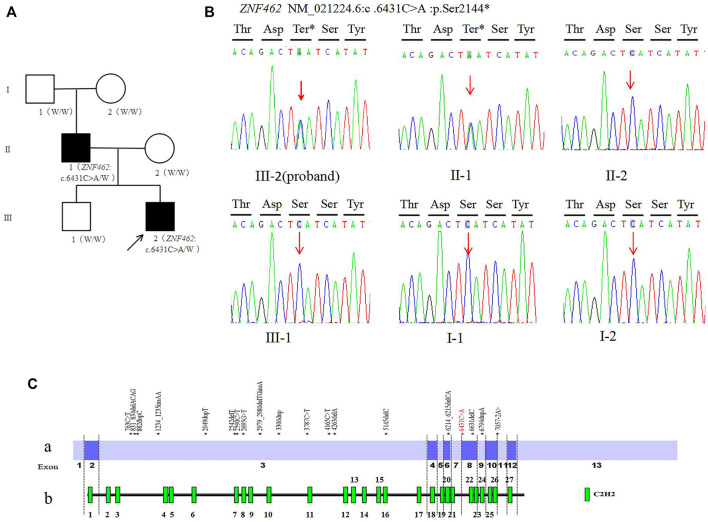
**(A)**. Family tree of this study (W: Wild type allele); **(B)**. Sanger sequencing of the *ZNF462:* c.6431C > A variant of family **(C)**. Distribution diagram of *ZNF462* gene variation (red fonts is the variant reported in this study) Ca. Distribution diagram of *ZNF462* gene variants reported in the HGMD database (numbers represent exons) Cb. Schematic diagram of C2H2 zinc finger structure distribution of ZNF462 protein.

The proband is a boy born prematurely (premature rupture of membranes at 36^+5^ weeks of gestation, G2P2, vaginal delivery, no asphyxia, Apgar score 10-10-10, and birth weight 3.08 kg). His parents are Chinese who are not close relatives. The child did not exhibit any abnormal breathing, vomiting, abdominal distension, or convulsions. His body temperature and reaction were normal, without the observance of yellow skin mucous membrane. His thoracic movements of both sides were the same, while the lungs were clear, the limbs were active, and the muscle tension was normal. He had no deformity in his skull, and the initial brain ultrasound showed that the triangular area of the bilateral ventricles had a slightly higher parenchymal echo. Furthermore, brain MRI showed no abnormalities. An echocardiogram detected a 1.2-mm patent ductus arteriosus. The boy exhibited an asymmetric crying face, and his left eyelid drooped significantly. The boy failed the hearing screening in the right ear. His father had a pathological droopy eyelid (the right eyelid) which was treated by surgery many years ago, while he has impaired hearing in the right ear. The proband’s mother, brother, and grandparents have no clinical symptoms (Figure 1A).

Furthermore, the chromosomal karyotyping of the proband was normal. It is normal to use tandem mass spectrometry for analysis of samples extracted from dried blood spots (DBS) collected from infants, and this test is used for the screening of amino acid metabolic disorders, organic acidemia, and fatty acid oxidative metabolic disorders by detecting the levels of dozens of amino acids, free carnitine, and acylcarnitine in samples extracted from newborn DBS. The genome copy number variation sequencing (CNV-seq) did not reveal a pathogenic CNV (pCNV), and the CNV-seq is a high-resolution genome-wide method to identify pCNV(>100 kb) based on low-coverage whole-genome sequencing. At 8 months old, the child manifested restriction in motor development and cannot sit alone or crawl with low muscle tone compared to his peers.

## 3 Method

### 3.1 Sample Collection

The study was approved by the Ethics Committee of Xi’an People’s Hospital (Xi’an Fourth Hospital), and the written informed consent was granted by the parents of the patient. In total, 3 ml of the peripheral blood sample was collected from the child, his parents, brother, and grandparents, respectively, and stored in EDTA anticoagulant tubes. The genomic DNA was extracted from all the blood samples using a QIAamp DNA Blood Mini Kit and stored at -20°C for subsequent usage.

### 3.2 Trio Whole-Exome Sequencing Analysis

The genomic DNA was analyzed by trio-WES. The NanoWES probe was used for the whole-exome DNA hybridization and enrichment in high-throughput sequencing (Nova Seq 6,000). Sequencing data analysis was conducted by the Verita Trekker® mutation site detection system and Enliven® mutation site annotation interpretation system. The analysis filtered out the variants with mutation frequencies greater than 1‰ in the human exon database (ExAC), the 1000 Genomes Project, and the Genome Aggregation Database (gnomAD),and also filtered the nonfunctional variation site (such as synonymous variants and noncoding region variants.). The pathogenicity prediction was performed using multiple software packages including SIFT, Polyphen2, and CADD. The potential pathogenic variant was determined along with the related disease database and relevant clinical reports.

### 3.3 Sanger Sequencing and Family Analysis

The pathogenic variant was detected using trio-WES and Sanger sequencing in the proband and his parents, and then Sanger sequencing validation was used for family analysis. The PCR amplicons of the target sequences were verified by 1% agarose gel electrophoresis, and sequencing was performed on the ABI 3500DX. The pathogenicity classification and data interpretation of the variations in the gene are based on the guidelines of the American Society of Medical Genetics and Genomics (ACMG) guidelines (Richards et al., 2015)

## 4 Results

### 4.1 Results of Genetic Analysis

The trio-WES analysis showed that both the proband and his father carried a novel heterozygous variant c.6431C > A (p.Ser2144*) in the *ZNF462* gene (NM_021224.6); however, no evidence of this variant was found in the other asymptomatic family members, including the proband’s mother, brother, and grandparents. The results were validated using Sanger sequencing (Figure 1B). So, for his father, c.6431C > A (p.Ser2144*) is *de novo* by both maternity and paternity confirmed (PS2_moderate). The variant is a nonsense variant in the coding region of the *ZNF462* gene, which may generate a premature stop codon and induce a loss-of-function effect (PVS1). The variant was not present in ExAC, 1,000G, and the gnomAD database (PM2_Supporting). According to the ACMG guidelines, the variant c.6431C > A (p.Ser2144*) should be classified as pathogenic (PVS1+PS2_moderate + PM2_Supporting).

### 4.2 Results of Literature Review

Related keywords to the “*ZNF462* gene” and “Weiss–Kruszka syndrome” were used during the search in the Chinese Journal Full-text Database (CNKI), Wanfang Data Knowledge Service Platform, and Weipu Database (covering time to May 2021), and no relative case was reported. The same keywords were used in PubMed (covering time to May 2021), and six publications were found containing cases of WSKA associated with the *ZNF462* gene. The first reported case identified a new balanced translocation t (2; 9) (p24; q32), and the multiple phenotypes of this individual could be due to the disruption in the *ZNF462* gene and *ASXL2* gene as a consequence of chromosomal rearrangement (Ramocki et al., 2003; Talisetti et al., 2003; Kruszka et al., 2019); Weiss *et al.* reported that the dysfunctional variants of *ZNF462* gene were found in six patients from four families with significant deletions in two different regions of chromosome nine were detected in two patients from two unrelated families (Weiss et al., 2017). Cosemans *et al.* reported a case of WSKA that was associated with chromosomal balanced translocation t (9; 13) (q31.2; q22.1) (Cosemans et al., 2018). Kruszka P *et al.* updated 14 cases of WSKA patients caused by the LOF variants in the *ZNF462* gene and characterized the clinical phenotypes for the studied cases (Kruszka et al., 2019). González–Tarancón R *et al.* demonstrated that a new frameshift variant in the *ZNF462* gene could be associated with WSKA syndrome (González–Tarancón et al., 2020). Recently, Iivonen AP *et al.* found a case with WSKA and Kallmann syndrome due to the deletion of chromosome 9q31.2, and Park *et al.* reported a case involving WSKA and empty sella syndrome (ESS) associated with the deficiency in the growth hormone (GHD) (Iivonen et al., 2021; Park et al., 2021). The clinical characteristics of the WSKA cases from the cited studies are summarized in Table 1.

**TABLE 1:** Clinical phenotypes of 29 patients and the family patients of this study caused by *ZNF462* gene mutation.

Patients	Sex	Age	Variant type	Inheritance	DD	Ptosis	Hypotonia	Ear malformation/Hearing loss	CHD	Down-slanting palpebral fissures	Arched eyebrows	Short upturned nose	Cupid’s bow	Epicanthal folds	Cranio-synostosis/Metopic ridging	Brain abnormalities	Feeding issues
1	M	16 months	c.2590C > T	Maternal (Mosaic)	+	+	+	+		−	+	−	+	+	−	−	+
			p.(Arg864*)														
2	M	10 years	c.2542del	*De novo*	+	+	−	−		+	+	+	+	+	−	−	+
			p.(Cys848Valfs*66)														
3	M	6 years	c.831_834del	*De novo*	+	−	+	+	+	−	−	+	−	−	−	−	+
			p.(Arg277Serfs*26)														
4	M	2 years	c.6214_6215del	*De novo*	+	+	−	+		−	−	−	+	+	+		+
		7 months	p.(His2072Tyrfs*8)														
5	F	14 years	c.763C > T	Paternal	+	+	−	+		−	+	−	−	+	+		−
			p.(Arg255*)														
6	F	7 months	c.7057–2A > G	*De novo*	+	+	+	+	+	+	+	+	+	+	−	+	+
7	M	13 years	c.6794dup	*De novo*	+	−	−	+		−	+	−	+	−	+		−
			p.(Tyr2265*)														
8	M	2 years	c.882dup	*De novo*	+	+	−	−		+	−	−	−	−	−	+	−
			p.(Ser295GLnfs*64)														
9	M	15 years	c.4165C > T	*De novo*	+	+	+	+		+	+	−	+	−	−		+
			p.(Gln1389*)														
10	M	8 years	c.1234_1235insAA	Unknown	+	+	-	+		−	−	−	−		−	−	+
			p.(Ser412*)														
11	F	2 years	c.6214_6215del	*De novo*	−	+	+			−	−	−	−		−		+
		5 months	p.(His2072Tyrfs*8)														
12	M	9 months	c.2049dup	*De novo*	+	+	+	−		+	+	+	+	+	−	−	−
			p.(Pro684Serfs*14)														
13	M	8 years	c.6631del	*De novo*	−	+	−	−		+	−	−	+	−	−		−
		7 months	p.(Arg2211GLyfs*59)														
14	F	8 years	c.2695G > T	Mother negative	+	−	+	−		−	+	+	−	−	−	−	+
			p.(Glu899*)	Father unknown													
15	F	2 years	c.3787C > T	Paternal	−	+	−	−		+	+	+	+	−	+	+	
			p.(Arg1263*)														
16	F	4 years	c.3787C > T	Paternal	−	+	−	−		+	−	−	+	+	+	−	
			p.(Arg1263*)														
17	M	34 years	c.3787C > T	Maternal	−	+	−	−		−	−	−	−	−	+		
			p.(Arg1263*)														
18	M	2 years	c.2979_2980delinsA	*De novo*	+	+	−	+		+	+	+	−	+	+	−	+
			p.(Val994Trpfs*147)														
19	M	32 months	c.4263del p.(Glu1422Serfs*6)	*De novo*	+	+	+	+	+	+	−	+	−	+	+	−	-
20	F	5 years	Chr9:g.(108940763-110561397)del (hg19)	*De novo*	−	−	+			+	+	+	+	+	−	+	
21	F	15 years	Chr9:g (108464368-110362345)del (hg19)	*De novo*	+	+	−		+	−	−	−	+	−	−		
22	M	9 years	c.5145delC	*De novo*	+	+	+	−		-	−	−	−	−	−	−	
			p.(Tyr1716Thrfs*28)														
23	F	5 years	t (2; 9) (p24; q32)	*De novo*	+	+	+	+	+	+	+	+	+	−	−	+	+
			disrupting ZNF462 and ASXL2														
24	M	24 years	t (9; 13) (q31.2; q22.1)	*De novo*	+	+	+	+		+	−	−	−	+	+	+	+
			disrupting ZNF462 and KLF12														
25	F	3 years	c.3306dup	*De novo*	+	+	+			+	−	−	−	+	−		+
		4 months	p.(Gln1103Thrfs*10)														
26	M	16 years	c.4185del	Mother negative	+	+	+	-	-	+	+	+	+	+	+	+	-
		9 months	p.(Met1396Ter)	Father unknown													
27	M	17 years	Chr9:g (108331353–	*De novo*	+	+	-	+	+			+				+	
		7 months	110707332)del (hg19)														
28	M	8 months	c.6431C > A	Paternal	+	+	+	+	+	−	−	−	−	−	−	−	−
			p.Ser2144*														
29	M	31 years	c.6431C > A	*De novo*	−	+	−	+		−	−	−	−	−	−		−
			p.Ser2144*														
Cohort prevalence					76%	86%	52%	51%	24%	52%	45%	41%	48%	45%	34%	28%	45%

Blank means no mention about the clinical features and/or no test results have been reported. Inheritance types were maternal 7% (2/29), paternal 14% (4/29), unknown 10% (3/29), *de novo* 69% (20/29).

Clinical characteristics were below: 76%(22/29) with DD, 86% (25/29) with ptosis, 52% (15/29) with hypotonia, 51%(15/29, six were hearing loss) with ear malformation/Hearing loss, 24%(7/29, 21 were not tested or not reported) with CHD, 52% (15/29) with down-slanting palpebral fissures, 45% (13/29) with arched eyebrows, 41% (12/29) with short upturned nose, 48% (14/29) with Cupid's bow, 45% (13/29) with epicanthal Folds, 34% (10/29) with metopic ridging, 52% (15/29) with hypotonia, 28% (8/29, 10 were not tested or not reported) with brain abnormalities and 45% (13/29) with feeding issues including our patient.

DD, developmental delay; CHD, congenital heart disease; MRI, magnetic resonance imaging; M, male; F, female.

Patient 1–26: Kruszka et al. (2019), Park et al. (2021), Patient 27: Iivonen et al. (2021), Patient 28: The proband of this study, Patient 29: The father of the patient 28.

In order to evaluate phenotype prevalence, we divided each positive phenotype report by the entire cohort (*n* = 29).

## 5 Discussion

The *ZNF462* gene, consisting of 13 exons, is located on chromosome 9q31.2. It encodes a protein (2,506 amino acids) with 27 C2H2 zinc finger structures, which participates in transcriptional regulation and the remodeling of the chromosome by bonding with DNA molecules (Nagase et al., 2001; Massé et al., 2010; Eberl et al., 2013). The zinc finger protein is highly conserved in most mammals. It is localized in the nucleus and widely expressed in various human tissues (Fagerberg et al., 2014). While the specific function of the protein has not been established, some studies on animal models demonstrated that *ZNF462* could play a vital role in embryonic development. For instance, the downregulation of *Zfp462* (*ZNF462*) gene expression in *Xenopus laevis* could interfere with early embryonic development by altering the cell division at the cleavage stage; however, this phenotype could be compensated through the introduction of additional human *ZNF462* mRNA (Laurent et al., 2009). In another study, the *Zfp462* knockout mice showed prenatal lethality and the heterozygous (*Zfp462* ^+/−^) mice developed diverse symptoms including low body weight, delayed brain weight development, anxiety-like behavior, and hair loss (Wang et al., 2017).

Given the evidence obtained from previous articles, the haploinsufficiency of the *ZNF462* gene is the genetic cause of WSKA. In clinical studies, WSKA is characterized by the mild and overall developmental delay with variable craniofacial abnormalities (typically ptosis, abnormal skull shape, lower oblique eyelid fissure, epicanthus, arched eyebrows, and short nose, etc.), while hypotonia and feeding difficulty are usually observed. Furthermore, a few cases reported dysplasia of the corpus callosum on brain imaging (Kruszka et al., 2019). By summarizing the existing cases (27 patients in the published reports and two patients from this study, in Table 1), it was found that ptosis, developmental delay, and autism are common manifestations in WSKA patients. Also, four out of the 27 patients had hearing impairments. Most patients were studied individually. Out of the reported cases, two cases resulted from the paternal inheritance, with a case resulting from the maternal inheritance, while another case was found to be due to maternal low-proportion mosaic (the mosaic ratio was 17%). This study discovered a new case with paternal inheritance in the Chinese population: the child had a paternal inheritance, but his father was *de novo*. They carried a novel nonsense variant c.6431C > A (p.S2144∗) in the *ZNF462* gene (NM_021224.6) found by using whole-exome sequencing, whereas the variant was not present in other tested family members. Both of them showed typical unilateral ptosis and unilateral hearing impairment. These findings indicated that the variant c.6431C > A (p.S2144∗) in the *ZNF462* gene could be associated with WSKA. The child showed a mild asymmetrical crying face during the neonatal period, and further had mild hypotonia and developmental delay. Pathogenic CNV was undetectable. Therefore, it is speculated that the observed clinical feature of WSKA could be individually specific.

By January 2021, the HGMD® database has recorded 24 *ZNF462* gene variants, including five nonsense variants, 12 frameshift variants, three missense variants, one splicing variant, two large fragment deletion variants, and one chromosome balanced translocation. These reported variants are mostly found in exon 3 of the *ZNF462* gene (Figures 1C,a), which may be related to exon 3 is the largest exon of the gene. The variant c.6431C > A (p.S2144∗) found in this study was located in exon 8 of the *ZNF462* gene, and there were several pathogenic nonsense variants reported in the downstream of c.6431C > A (p.S2144∗). This point further confirms the pathogenicity of the variant. In addition, the variant may undergo nonsense-mediated decay (NMD), which may lead to heterozygous loss of *ZNF462* transcript and consequently result in the disease phenotype. Moreover, the variant c.6431C > A (p.S2144∗) was located in the region between the 21st and 22nd C2H2 zinc finger structures, and it causes amino acid deletion from amino acids 2,144 (Figure 1C,b), leading to the absence of the last six zinc finger structures. Hence, this variant was assumed to be responsible for DNA binding impairment and the subsequent protein dysfunction, which needs to be investigated in subsequent studies.

This is the first pedigree of WSKA in China. A novel nonsense variant c.6431C > A (p.S2144∗) in the *ZNF462* gene was identified in the proband and his father; this finding enriched the variant spectrum of the *ZNF462* gene. The proband and his father showed unilateral ptosis and unilateral hearing impairment which were typical symptoms of WSKA (Table 1), so they were diagnosed combined with the sequencing result. The mild asymmetrical crying face during the neonatal period only showed in the proband could be individually specific, and the role of the pathogenic variant in this case required further investigation. The inheritance type of the proband was paternal, and *de novo* mutations were still the main way of inheritance in all the reported WSKA cases (Table 1). This study provided more clinical and genetic evidence to support the haploinsufficiency of the *ZNF462* gene proposed by earlier studies. The novel variant and phenotypes seen in this family contributed to understanding the clinical features, genetic characteristics, and diagnostic protocols for WSKA.

While WES has facilitated the identification of pathogenic gene variants for many rare diseases (Yang et al., 2014), the increasing knowledge will improve the diagnosis accuracy of rare diseases and contribute to the prediction or the prevention of birth defects. Combined with trio-WES analysis, the patients in this study were finally diagnosed. Besides the traditional diagnostic approach, the introduction of trio-WES can lead to the effective identification and differentiation of the variants, and thus offer feasible support for clinical diagnosis and treatment.

## Data Availability

The datasets presented in this study can be found in online repositories. The names of the repository/repositories and accession number(s) can be found below: CNGB Sequence Archive (CNSA) of China National GeneBank DataBase (CNGBdb); accession number CNP0002511.

## References

[B1] Cosemans N. Vandenhove L. Maljaars J. Van Esch H. Devriendt K. Baldwin A. (2018). *ZNF462* and *KLF12* Are Disrupted by a De Novo Translocation in a Patient with Syndromic Intellectual Disability and Autism Spectrum Disorder. Eur. J. Med. Genet. 61 (7), 376–383. 10.1016/j.ejmg.2018.02.002 29427787

[B2] Eberl H. C. Spruijt C. G. Kelstrup C. D. Vermeulen M. Mann M. (2013). A Map of General and Specialized Chromatin Readers in Mouse Tissues Generated by Label-free Interaction Proteomics. Mol. Cel 49 (2), 368–378. 10.1016/j.molcel.2012.10.026 23201125

[B3] Fagerberg L. Hallström B. M. Oksvold P. Kampf C. Djureinovic D. Odeberg J. (2014). Analysis of the Human Tissue-specific Expression by Genome-wide Integration of Transcriptomics and Antibody-Based Proteomics. Mol. Cell Proteomics 13 (2), 397–406. 10.1074/mcp.M113.035600 24309898 PMC3916642

[B4] González-Tarancón R. Salvador-Rupérez E. Miramar Gallart M. Barroso E. Díez García-Prieto I. Pérez Delgado R. (2020). A Novel Mutation in the *ZNF462* Gene c.3306dup; p.(Gln1103Thrfs*10) Is Associated to Weiss-Kruszka Syndrome. A Case Report. Acta Clinica Belgica 1, 1–4. 10.1080/17843286.2020.1780391 32543299

[B5] Iivonen A.-P. Kärkinen J. Yellapragada V. Sidoroff V. Almusa H. Vaaralahti K. (2021). Kallmann Syndrome in a Patient with Weiss-Kruszka Syndrome and a De Novo Deletion in 9q31.2. Eur. J. Endocrinol. 185 (1), 57–66. 10.1530/eje-20-1387 33909591 PMC8183635

[B6] Kruszka P. Hu T. Hong S. Signer R. Cogné B. Isidor B. (2019). Phenotype Delineation of *ZNF462* Related Syndrome. Am. J. Med. Genet. 179 (10), 2075–2082. 10.1002/ajmg.a.61306 31361404 PMC6935050

[B7] Laurent A. Masse J. Omilli F. Deschamps S. Richard-Parpaillon L. Chartrain I. (2009). ZFPIP/Zfp462 Is Maternally Required for Proper Early *Xenopus laevis* Development. Developmental Biol. 327 (1), 169–176. 10.1016/j.ydbio.2008.12.005 19111535

[B8] Massé J. Laurent A. Nicol B. Guerrier D. Pellerin I. Deschamps S. (2010). Involvement of *ZFPIP/Zfp462* in Chromatin Integrity and Survival of P19 Pluripotent Cells. Exp. Cel Res. 316 (7), 1190–1201. 10.1016/j.yexcr.2010.02.024 20219459

[B9] Nagase T. Nakayama M. Nakajima D. Kikuno R. Ohara O. (2001). Prediction of the Coding Sequences of Unidentified Human Genes. XX. The Complete Sequences of 100 New cDNA Clones from Brain Which Code for Large Proteins *In Vitro* . DNA Res. 8 (2), 85–95. 10.1093/dnares/8.2.85 11347906

[B10] Park J. Ha D. J. Seo G. H. Maeng S. Kang S. M. Kim S. (2021). Empty Sella Syndrome Associated with Growth Hormone Deficiency: the First Case Report of Weiss-Kruszka Syndrome. J. Korean Med. Sci. 36 (18), e133. 10.3346/jkms.2021.36.e133 33975400 PMC8111047

[B11] Ramocki M. B. Dowling J. Grinberg I. Kimonis V. E. Cardoso C. Gross A. (2003). Reciprocal Fusion Transcripts of Two Novel Zn-finger Genes in a Female with Absence of the Corpus Callosum, Ocular Colobomas and a Balanced Translocation between Chromosomes 2p24 and 9q32. Eur. J. Hum. Genet. 11 (7), 527–534. 10.1038/sj.ejhg.5200995 12825074

[B12] Richards S. Aziz N. Bale S. Bick D. Das S. Gastier-Foster J. (2015). Standards and Guidelines for the Interpretation of Sequence Variants: a Joint Consensus Recommendation of the American College of Medical Genetics and Genomics and the Association for Molecular Pathology. Genet. Med. 17 (5), 405–424. 10.1038/gim.2015.30 25741868 PMC4544753

[B13] Talisetti A. Forrester S. R. Gregory D. Johnson L. Schneider M. C. Kimonis V. E. (2003). Temtamy-like Syndrome Associated with Translocation of 2p24 and 9q32. Clin. Dysmorphol. 12 (3), 175–177. 10.1097/01.mcd.0000072161.33788.56 14564155

[B14] Wang B. Zheng Y. Shi H. Du X. Zhang Y. Wei B. (2017). *Zfp462* Deficiency Causes Anxiety-like Behaviors with Excessive Self-Grooming in Mice. Genes Brain Behav. 16 (2), 296–307. 10.1111/gbb.12339 27621227

[B15] Weiss K. Wigby K. Fannemel M. Henderson L. B. Beck N. Ghali N. (2017). Haploinsufficiency of *ZNF462* Is Associated with Craniofacial Anomalies, Corpus Callosum Dysgenesis, Ptosis, and Developmental Delay. Eur. J. Hum. Genet. 25 (8), 946–951. 10.1038/ejhg.2017.86 28513610 PMC5567153

[B16] Yang Y. Muzny D. M. Xia F. Niu Z. Person R. Ding Y. (2014). Molecular Findings Among Patients Referred for Clinical Whole-Exome Sequencing. JAMA 312 (18), 1870–1879. 10.1001/jama.2014.14601 25326635 PMC4326249

